# Revisiting volatile organic compounds’ role in plant communication using real-time bioimaging

**DOI:** 10.3389/fpls.2026.1829872

**Published:** 2026-06-18

**Authors:** Natsuko Kinoshita, Mariko S. Hirakawa, Takuya Uehara, Barry Lustig

**Affiliations:** 1 Faculty of Life and Environmental Sciences, University of Tsukuba, Tsukuba, Ibaraki, Japan; 2 Division of Insect Advanced Technology, Institute of Agrobiological Sciences, NARO, Tsukuba, Ibaraki, Japan

**Keywords:** wide-field non-invasive real-time fluorescent imaging, plant communication, green leaf volatile (GLV), volatile organic compound (VOC), herbivore-induced plant volatile (HIPV), calcium signal, anti-herbivory insect stress responsive gene expression, Arabidopsis

## Abstract

Plants release Volatile Organic Compounds (VOCs) in response to insect attacks. VOC facilitates communication with neighboring, undamaged plants. In response to VOC from insect damaged plants, neighboring undamaged plants upregulate their own defenses as if they were being attacked themselves. To date, Green Leaf Volatiles (GLVs) within VOC have been widely considered a primary mediator for plant communication. GLV is a six-carbon compound which all land plants emit immediately and in large quantities after wounding. We hypothesized that GLVs’ lack of specificity and abundance is unlikely to account for key aspects of plant communication like increased sensitivity between closely related plants. To test our hypothesis, we used an Arabidopsis accession which does not produce GLVs. We also developed a non-invasive imaging technique to visualize plant communication utilizing expressions of insect stress marker gene *VSP1*. Our analysis confirmed that plant communication occurs even without GLVs. Cytosolic calcium ion concentration increased before this timing, and moved towards the tip of the leaf in undamaged plants. Additionally, when plants were damaged by insects, acetophenone and alkanes accumulated the experiment’s enclosed space. This suggests that plants communicate independently of GLV using alkanes and acetophenone, which are known to attract natural enemies of herbivore insects like parasitoid wasps.

## Introduction

Plants release smell, or Volatile Organic Compounds (VOC), when insects attack (Schuman and Baldwin, 2016; Turlings and Erb, 2018; Erb and Reymond, 2019; Brosset and Blande, 2021). Nearby undamaged plants recognize this smell and activate their own defenses accordingly (Karban et al., 2006; Karban et al., 2014; Schuman and Baldwin, 2016; Erb and Reymond, 2019; Karban, 2021; Gong et al., 2023; Arimura and Uemura, 2024).

For the purposes of this paper, plants releasing VOC as they are being attacked by herbivore insects are *Emitter* plants.

Undamaged plants detecting VOC released from their neighboring plants under herbivore insect attack are *Receiver* plants.

In *Emitter plants*, cytosolic calcium ion concentration ([Ca^2+^]_cyt_) elevates at the site-of-injury and propagates throughout their vascular system. When calcium waves reach distant tissues within a plant, they activate defense responses by inducing the expression of enzymes involved in jasmonic acid (JA) biosynthesis, a critical phytohormone that mediates resistance to insect attack (Mousavi et al., 2013; Zhang et al., 2017; Erb and Reymond, 2019). The resulting increase in JA promotes the expression of defense related genes like *JAZ10* and *VEGETATIVE STORAGE PROTEIN1 (VSP1)*, an established marker gene for insect stress (Berger et al., 2002; Mousavi et al., 2013). These long-distance calcium and defense signaling processes are dependent on ion channel encoding *GLUTAMATE RECEPTOR-LIKE (GLR)* (Mousavi et al., 2013; Toyota et al., 2018).

In *Receiver* plants, exposure to VOC from nearby, insect damaged plants triggers the activation of their own JA biosynthesis and JA-dependent defense pathways. For example, when leaves of lima bean *Emitter plants* (*Phaseolus lunatus* cv. Sieva) were infested with spider mites (*Tetranychus urticae*), neighboring lima bean *Receiver* plants upregulated mRNA level of *LIPOXYGENASE*, a key enzyme in JA biosynthesis (Arimura et al., 2000). Similarly, when lima bean *Emitter* plants were attacked by leafminers (*Liriomyza huidobrensis*), adjacent *Arabidopsis Receiver* plants exhibited increased *VSP1* transcripts, which suggests a corresponding boost in their defense responses (Zhang et al., 2017).

The current view on communication between *Emitter* and *Receiver* plants is that communication facilitated primarily by Green Leaf Volatiles (GLVs) (Aratani et al., 2023). GLVs are six-carbon volatile compounds, including aldehydes, alcohols and their corresponding esters. They are produced by all terrestrial green plants in large quantities when plants are damaged. It is also known that plants with closer genetic relationships communicate with higher sensitivity (Karban et al., 2013). Therefore we explored whether plants could communicate effectively without GLVs. Our findings indicate that GLVs are not a primary determinant of plant communication. This suggests that additional, more specific volatile cues underlie the facilitation of plant communication.

## Results and discussion

### VOC emitted from mutants lacking GLV activates insect defenses in nearby plants

**Figure 1:**
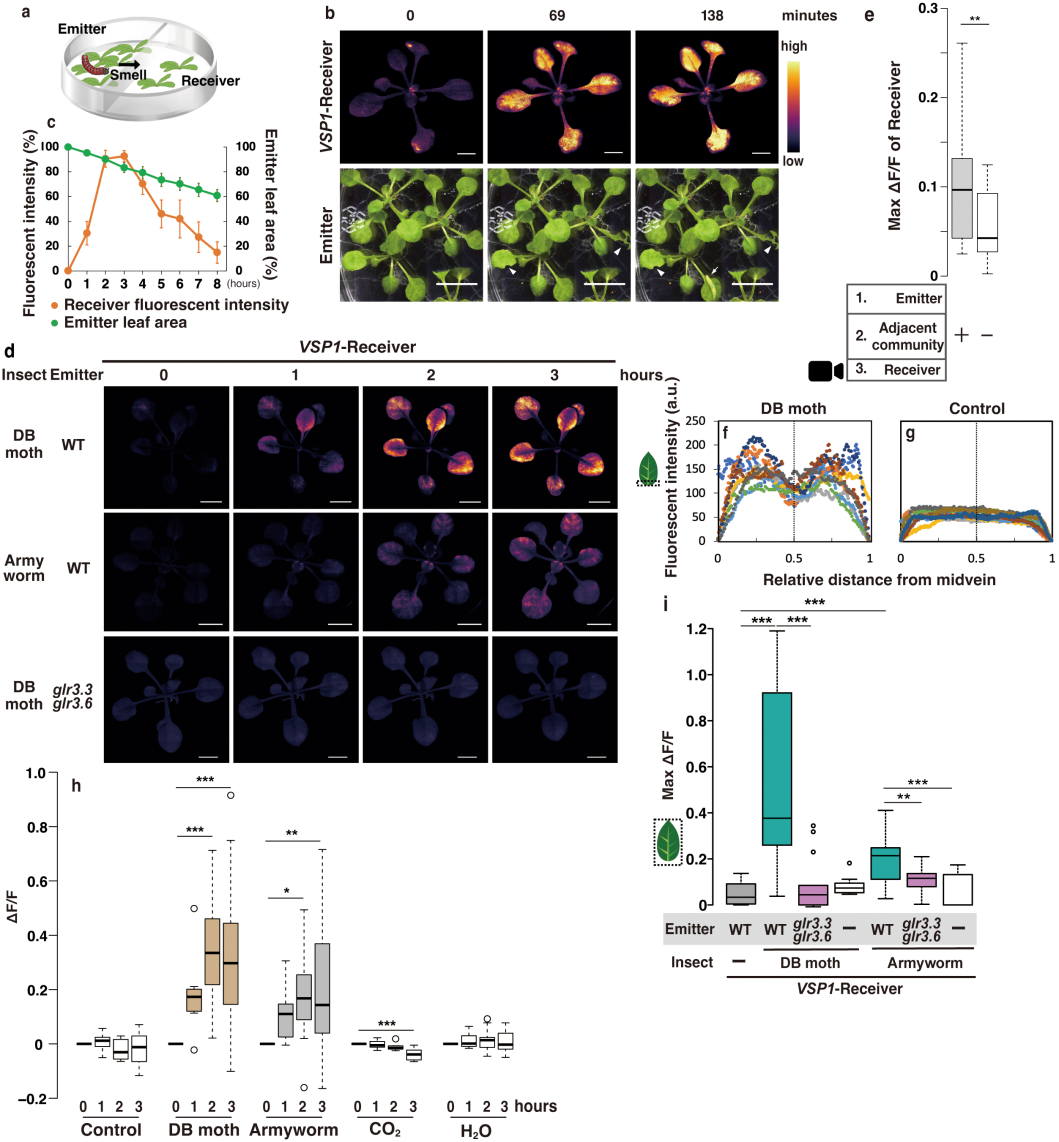
*Receiver* detects insect damage without GLV. **(a)** Experimental setup. Whole Arabidopsis plants were grown inside a Petri dish and used as *Emitter* plants. At the time of the experiment, insects were placed on one side of the Petri dish separated by a divider. On the opposite side, whole Arabidopsis plants were grown as *Receiver.* No insects were added until the start of each experiment. We grew plants inside the dish on media to reduce plant stress (as was done for all other experiments). **(b)** Top panel, *VSP1-Receiver* emits fluorescent signal in response to VOC from diamondback moth (DB moth)*-*damaged *Emitter*. Scale bar: 5 mm. Bottom panel, DB moth-damaged *Emitter* under white light from the same experiment. Scale bar: 1 cm. Arrow indicates presence of DB moth; triangles indicate leaf damage from DB moth. **(c)** Quantification of experiments represented in **(b)**. Relative fluorescent intensities (%) were calculated as maximum F/F0 as 100%. Bars indicate s.e.m. n = 5 **(d)** Insect damaged *Emitter* induces fluorescent signal in *VSP1-Receiver*. *VSP1-Receiver* response to DB moth-damaged WT *Emitter* (top), armyworm-damaged WT *Emitter* (middle), and DB moth-damaged *Emitter* with double mutations in *GLUTAMATE RECEPTOR-LIKE GENES* (*glr3.3glr3.6*) (bottom). Scale bar: 5 mm. **(e)** Quantification of fluorescent signal intensity using a three-way split plate. In the first section of the split plate, we grew WT *Emitter.* DB moth was added after the first frame was recorded. In the second section, we grew fifteen WT plants. In the third section, we grew *VSP1-Receiver*. Fluorescent signals from *VSP1*-*Receiver* in the third section were analyzed continuously using time-lapse imaging. n ≧ 10 **(f, g)** Fluorescent signal intensity is plotted across a horizontal section of *VSP1-Receiver* responding to WT Arabidopsis damaged by DB moth **(f)** control **(g)**, at the leaf base. 11-leaves **(f)** and 12-leaves **(g)** are shown with a different color for each leaf. **(h)** Quantification of *VSP1-Receiver* signal over time in response to DB moth-damaged WT *Emitter* (tan), armyworm-damaged WT *Emitter* (gray), and undamaged-WT *Emitter* (white), carbon dioxide, and extra moisture. n ≧ 7 **(i)** *VSP1-Receiver* requires *Emitter* genes *GLR3.3* and *GLR3.6* to respond to DB moth- and armyworm-damaged *Emitter.* Values of maximum F/F0 of *VSP1-Receiver* are shown for: experiments using WT *Emitter* with insects (green); WT *Emitter* without insects (gray); *Emitter* with *glr3.3glr3.6* background (magenta); and without *Emitter* (insects only, white). n ≧ 8 **(e, h, i)** Center lines show medians; box limits indicate 25th and 75th percentiles; whiskers extend 1.5 times interquartile range; and outliers are represented by dots. *p < 0.05, **p < 0.01, ***p < 0.001, using Dunnett’s *post hoc* multiple comparison test.

We developed a noninvasive experimental setup to visualize VOC-mediated plant communication. Petri dishes were separated in half using a porous barrier which enables air to move freely within the petri dish. Insects were only able to eat plants on one side of the dish. On half of the dish, we grew five *Emitter* plants for two weeks. Insects were added at the time of the experiment. On the other half of the dish, we grew two *Receiver* plants (**Figure 1a**). Fluorescence was analyzed continuously using time-lapse imaging.

To visualize VOC-mediated communication between plants, we used insect infested Wild-type (WT) Arabidopsis as *Emitter* plants. For *Receiver* plants, we used transgenic Arabidopsis containing *Yellow Fluorescent Protein* (*YFP*) fused to the promoter of *VSP1* (*VSP1-Receiver*) (Betsuyaku et al., 2018; Kinoshita and Betsuyaku, 2018; Kinoshita et al., 2019). Arabidopsis Columbia accession (Col-0) for both *Emitter* and *Receiver* plants were used. Col-0 accessions do not produce detectable amounts of GLVs because of their lack of functional HYDROPEROXIDE LYASE (HPL), a key enzyme in GLV synthesis (Duan et al., 2005).

When *Emitter* plants were infested with diamondback moth (DB moth) (*Plutella xylostella*), a specialist caterpillar for the Brassicaceae family which includes Arabidopsis, *VSP1-Receiver* displayed an intense fluorescent signal (**Figures 1b-d**; **Supplementary Figure 1**; **Supplementary Movies S1**, **S2**). Fluorescence was detectable at the onset of an insect attack indicated by initial decline of surface area exhibiting chlorophyll autofluorescence (**Figures 1b, c**; **Supplementary Figure 2**). For example, one hour after DB moth infestation, less than five percent of *Arabidopsis-Emitter* leaves were damaged (**Figure 1c**). Surprisingly, *VSP1-Receiver* displayed approximately 30% of peak fluorescence at this early timing (**Figure 1b-c**). There was also greater *VSP1-Receiver* signal intensity when the number of intact plants increased suggesting signaling amplification within groups of plants (**Figure 1e**).

Fluorescent intensities were measured and plotted along the horizontal section of the leaf base to analyze signal distribution within *VSP1-Receiver*. Fluorescence declined around the midvein but was highly expressed in the tissue surrounding the midvein (**Figures 1f, g**). This suggested that insect defense in the lamina tissue surrounding midvein was activated within *Receiver* plants via the detection of VOC (Caldwell et al., 2015). In a parallel experiment, WT Arabidopsis was subjected to polyphagous oriental armyworm (armyworm) (*Mythimna separata)*. This was done to demonstrate whether the *VSP-1* expression pattern we observed using DB moth was specific to specialist herbivore insects.

We found that while *VSP1-Receiver* plants displayed an increase of fluorescence when exposed to armyworm-infested *Emitter* plants, its signal was weaker when compared to the DB moth experiments (**Figures 1d, h, i**; **Supplementary Figure 1**; **Supplementary Movie S3**). It is likely that relatively large armyworms consumed *Emitter* plants faster than they could release sufficient quantities of VOC. In any case, the signal followed a similar pattern to that of DB Moth. To ensure that these signals were not caused by the respiration of insects, we verified that neither carbon dioxide nor extra humidity induced a clear fluorescent signal (**Figure 1h**). There was a slight decrease of fluorescence in response to increased carbon dioxide. Insect respiration likely dampens rather than induces signal. We also confirmed that fluorescent signals are detected even when holes were made in the Petri dish to accelerate ventilation (**Supplementary Figure 3**).

### *GLR*s in damaged *Emitter plant* trigger insect defenses in *Receiver* plants

*GLUTAMATE RECEPTOR-LIKE proteins (GLR*s) are necessary to express JA-inducible anti-herbivory genes in plants under insect attack (Mousavi et al., 2013; Toyota et al., 2018). So, we sought to test whether *GLR* genes in *Emitter* plants are required to release VOC.

When double mutant *glr3.3glr3.6* was used as an *Emitter* plant, *VSP1-Receiver* plants exhibited reduced fluorescence when compared to the WT *Emitter* experiments (**Figures 1d, i**; **Supplementary Figure 1**). Fluorescent signal from *VSP1-Receiver* did not elevate when exposed to the scent of herbivore insects by itself (**Figure 1i**). *Emitter* genes *GLR3.3* and *GLR3.6* were required to communicate with *Receiver* plants.

### Calcium ion movement in *Receiver* is triggered by VOC blends lacking GLVs

**Figure 2:**
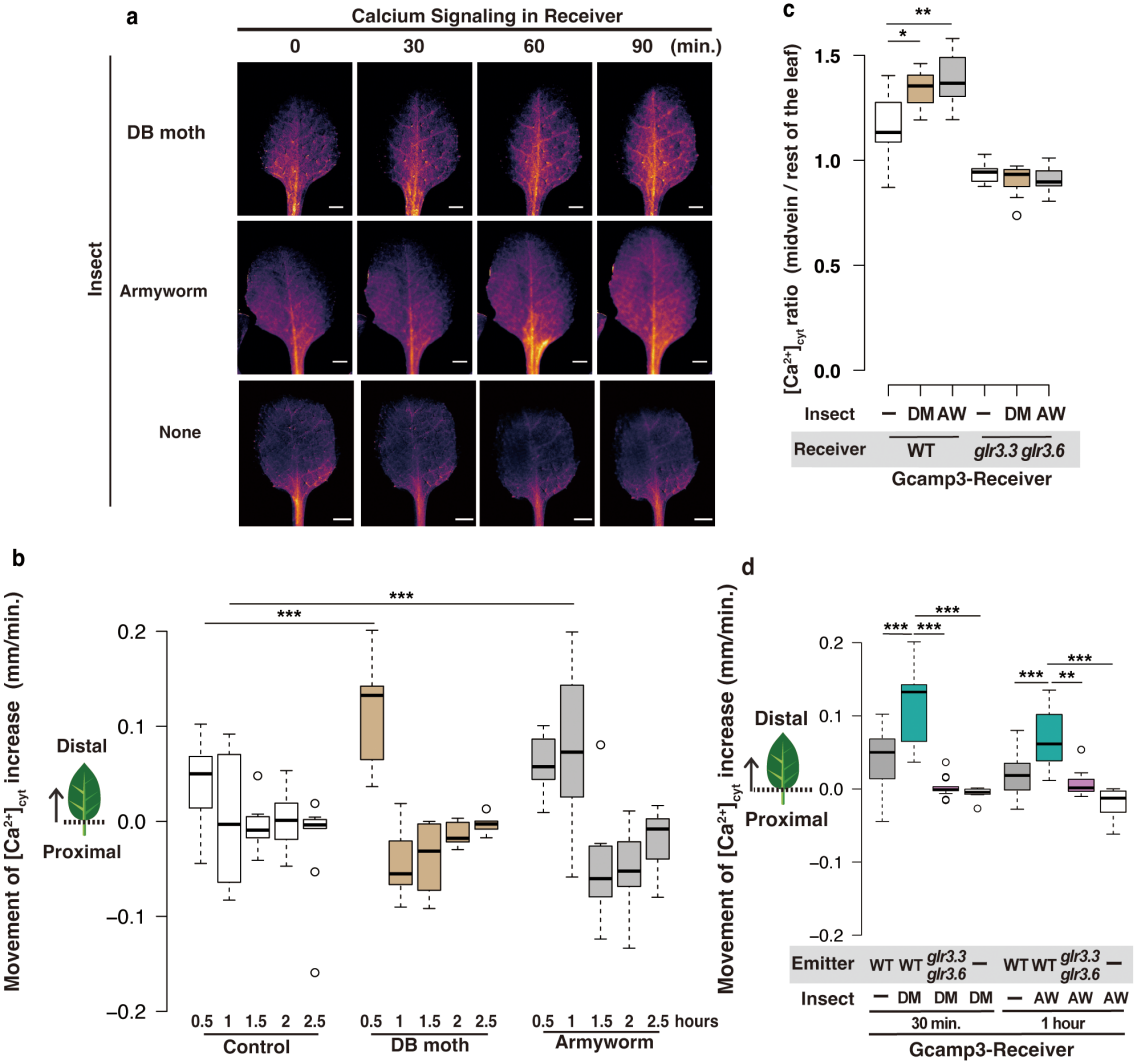
Calcium signal activates in *Receiver* without GLV. **(a)** [Ca^2+^]_cyt_ increase in *Receiver* responding to VOC from Wild-type (WT) *Emitter* damaged by diamondback moth (DB moth) (top), armyworm (middle), and control (bottom). Scale bar: 1 mm. **(b)** Area of increased *Receiver* [Ca^2+^]_cyt_ extends along the midvein when *Emitter* is damaged by diamondback moth (DB moth, middle, tan) or armyworm (right, gray) but not when *Emitter* is undamaged (left, white). n ≧ 10 **(c)** *Receiver* [Ca^2+^]_cyt_ increase occurs predominantly in the midvein and is dependent on *GLR*s. Ratios of *Receiver* [Ca^2+^]_cyt_ increase in the midvein compared to the rest of the leaf are shown for WT *Receiver* (left) and *glr3.3glr3.6 Receiver* (right). Control with no insect (white); DB moth (tan); armyworm (gray) are shown. n ≧ 7 **(d)** *GLR* genes in *Emitter* are necessary for the movement of [Ca^2+^]_cyt_ in *Receiver.* Length of increased [Ca^2+^]_cyt_ region on midvein was measured 30-minutes after insects were added. Experiments using WT *Emitter* with insects (green); WT *Emitter* without insects (gray); *Emitter* with *glr3.3glr3.6* background (magenta); and insects only (white) are shown. n ≧ 8 **(b-d)** Center lines show medians; box limits indicate 25th and 75th percentiles; whiskers extend 1.5 times interquartile range; and outliers are represented by dots. *p < 0.05, **p < 0.01, ***p < 0.001, using Dunnett’s *post hoc* multiple comparison test.

Prior studies demonstrate that plants increase their [Ca^2+^]_cyt_ levels after exposure to terpenoids and Green Leaf Volatiles, GLVs (Hu et al., 2019; Wang and Erb, 2022; Aratani et al., 2023). We examined whether natural VOC without GLV from plants under herbivore insect stress increase calcium waves in *Receiver* plants. Using time-lapse imaging, we visualized [Ca^2+^]_cyt_ increase in *Receiver* using Arabidopsis expressing GCaMP3 (*Gcamp3-Receiver*), a protein-based fluorescent calcium sensor (Toyota et al., 2018). Fluorescent signal was detectable in the midvein and extended toward the tip of the leaf in *Receiver* plants (**Figures 2a, b**). For both DB moth and armyworm, this corresponded with previous findings that [Ca^2+^]_cyt_ travels through the *Emitter* plant’s vascular system (Toyota et al., 2018). *Receiver* plants appear to mirror the *Emitter* response to insect damage without being physically damaged.

We quantified the speed of [Ca^2+^]_cyt_ increase by measuring length of the signal increase along the midvein from the base of the leaf. [Ca^2+^]_cyt_ increase moved markedly (more than two orders of magnitude) more slowly than what was observed in *Emitter* plants. The peak speed of [Ca^2+^]_cyt_ increase was 118 ± 15 μm/min. within a *Receiver* plant’s leaf in response to DB Moth damaged *Emitter* plants (**Figure 2b**). In *Emitter* plants, [Ca^2+^]_cyt_ increase moved approximately 6 mm/min when Arabidopsis was damaged by another lepidopteran insect, cabbage butterfly (*Pieris rapae)* (Toyota et al., 2018). *Emitter* plants appear to respond faster than *Receiver* plants to warn its own leaves of imminent insect threats.

### *Receiver*-*GLRs* are required for calcium signaling in *Receiver* plants

*Emitter*-*GLR*s are required for the upregulation of fluorescent signal in *VSP1*-*Receiver* (**Figure 1i**) as well as the production of calcium signals in *Emitter* plants (Nguyen et al., 2018; Toyota et al., 2018). VOC from infested Wild-type (WT) *Emitter* plants did not increase fluorescent signal around the midvein of *Gcamp3-Receiver/glr3.3glr3.6* (**Figure 2c**; **Supplementary Figure 4**). This confirms that *Receiver GLR3.3* and *GLR3.6* are necessary to activate calcium signaling in the *Receiver* plants. GLR is localized within vascular bundles (Toyota et al., 2018). It is likely that VOCs enter plants through the stomata (Aratani et al., 2023), and diffuse across mesophyll cells which encase vascular bundles where GLRs are expressed (Nguyen et al., 2018). Additionally, VOC likely interacts with proteins expressed in guard and/or mesophyll cells. These proteins activate GLR expressed in contact cells in xylem parenchyma and phloem where calcium signals originate (Toyota et al., 2018). For instance, an analog of the terpene caryophyllene interacts with the transcriptional repressor TOPLESS (TPL) (Nagashima et al. 2019). Accordingly, *TPL* and *TPR4* (*TPL RELATED 4*) are highly expressed in stomata guard and mesophyll cells (Winter et al., 2007).

### *Emitter GLRs* are necessary for *Receiver* plant calcium signaling

*Emitter GLRs* are necessary to upregulate *VSP1* expression in *Receiver* plants (**Figures 1b, h, i**). When *Emitter* plants contained *glr3.3* and *glr3.6* mutations, the region of [Ca^2+^]_cyt_ increase in *Receiver* plants did not extend toward the tip of the leaf when compared to WT *Emitter* (**Figure 2d**, **Supplementary Figure 5**). *Emitter GLR*s are therefore necessary to produce calcium waves in *Receiver* plants.

### Acetophenone and alkanes are mediators of VOC-mediated communication

**Figure 3:**
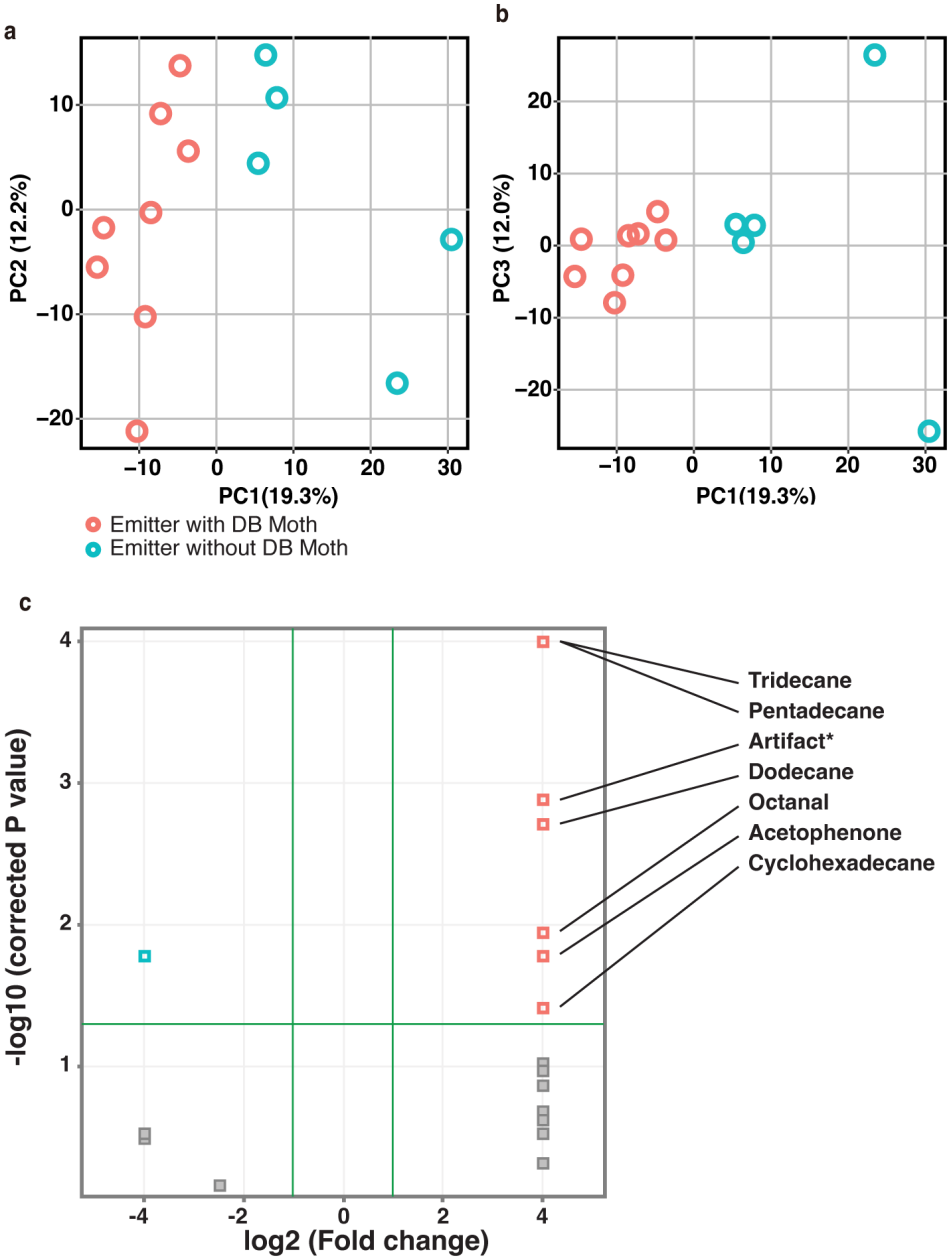
VOC from plant groups with insect-infested *Emitter* plants accumulated hydrocarbons and acetophenone. Principal components (PC) between PC1 and PC2 **(a)**, and PC1 and PC3 **(b)** are shown. Volcano plot of components detected in groups of plants with *Emitter* infested by DB moth and control **(c)**. Red and blue symbols show VOC components in plant groups infested with DB moth and control. *silyl-derived compound from silica monolith adsorbents.

We analyzed how plant communication occurs without GLVs by isolating chemical compounds found in VOC using gas chromatography mass spectrometry (GC-MS). The median number of detected compounds was 22 (range 14–24) in damaged *Emitter* plants, compared to 10 (range 6–13) in undamaged controls. Principal component analysis clarified that biological replicates clustered tightly (**Figures 3a, b**). Volcano plot analysis identified chemical compounds whose levels changed significantly in close proximity to damaged *Emitter* plants (**Figure 3c**). Among these, 23-chemical components increased significantly when plants were infested with DB moth (**Supplementary Table 1**). GLVs like (E)-2-hexenal, (Z)-3-hexenal, and (Z)-3-hexenyl acetate were absent due to their lack of functional Hydroperoxide Lyase (HPL), the enzyme responsible for GLV synthesis (Duan et al., 2005).

Octanal, an aldehyde known to attract the natural enemies of herbivore insects, was detected in our analysis (**Figure 3c**). For example, octanal is released by cabbage when infested with pierid butterfly larvae (Birkett et al., 2003). Hydrocarbons like tridecane, pentadecane, dodecane, cyclohexadecane were also found (**Figure 3c**). These hydrocarbons were detected as a part of VOC emissions in various plant species, inclusive of rice and tomato (Errard et al., 2015; Yi et al., 2023; Gokila et al., 2024; Yasa et al., 2024). While these VOC were detected in other plant species, their significance was likely missed in terms of their function as a facilitator of plant communication under duress from herbivore insects.

Notably, acetophenone, a major compound found in Alyssum (*Lobularia maritima*) (Brassicaceae) was identified (**Figure 3c**). Volatiles from Alyssum attract *Cotesia vestalis*, a parasitoid wasp and natural enemy of DB moth (Zubkov and Kouznetsov, 2023). Acetophenone is known to extend *C. vestalis’* lifespan, and increase *C. vestalis*’ parasitism rate (Chen et al., 2020). Acetophonone enables Brassicaceae plants to provide an important benefit to *Cotesia vestalis* without extending these same benefits to DB moths. Further investigation is needed to clarify whether these compounds play a direct role in triggering the GCaMP3 calcium waves and VSP1-YFP expression.

Emission of octanal, four aforementioned hydrocarbons, and acetophenone required *GLRs.* Volcano plot analysis using *GLR* mutants did not detect significant differences between *Emitter* plants with and without DB moth samples (p < 0.05, fold change ≥ 2, **Supplementary Table 2**). Although relationships between alkane biosynthesis and GLR remain unclear, acetophenone is produced via β-oxidative pathway (Zhai et al., 2025). Octanal is derived from lipid oxidation (Liang et al., 2022). At the same time, GLR triggers jasmonic acid biosynthesis and this requires both LOX-mediated lipid oxidation and subsequent β-oxidation steps (Li et al., 2005; Toyota et al., 2018). It is plausible that GLR-dependent signaling promotes the accumulation of both compounds by modulating these metabolic activities although direct regulatory links are not yet established.

## Conclusion

**Figure 4:**
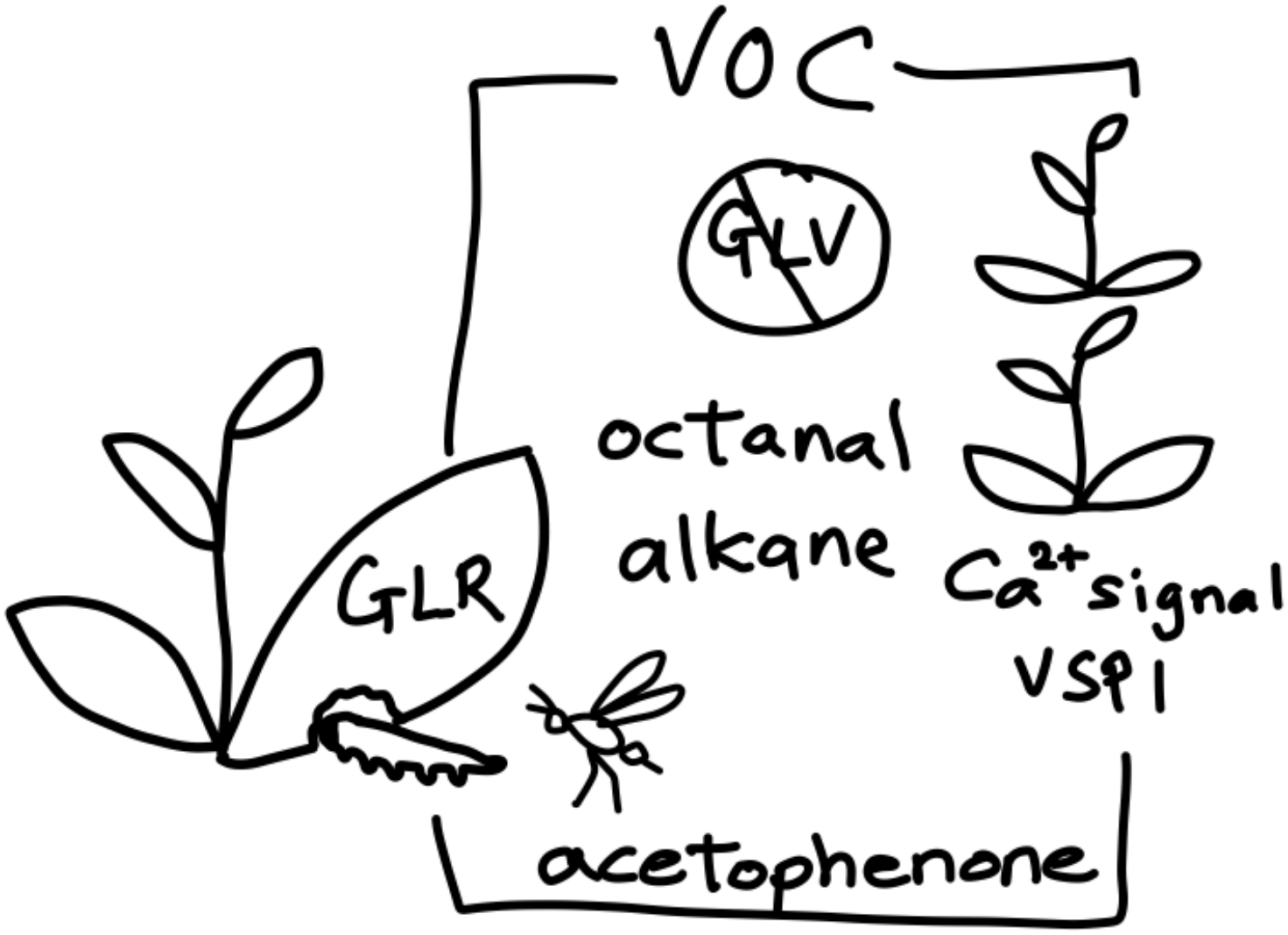
Model of plant resistance to insect attack without GLVs. After herbivore damage, (1) *Emitter* releases VOC including octanal, and hydrocarbons, acetophenone in an GLR dependent manner. (2) In response, *Receiver* defense is activated as observed in calcium signal activation and the expression of the defense marker gene *VSP1*. Acetophenone released by *Emitter* attracts parasitoid wasps, a natural enemy of herbivore insects, and also prolongs wasp lifespan.

We established a technique to analyze VOC mediated plant-to-plant communication by using non-invasive time-lapse imaging. Even under conditions where Green Leaf Volatiles (GLVs) are absent, VOCs emitted from *Emitter* plants are sufficient to activate defense responses in *Receiver* plants (**Figure 4**). GLRs in *Emitter* plants are necessary to emit specific VOCs, including hydrocarbons, and acetophenone, as well as for the subsequent upregulation of defense-related molecular markers in *Receiver* plants. In sum, basic assumptions about how plant communication works at a molecular level may be fruitfully examined as new techniques make it possible to do so. As our knowledge about the molecular mechanisms of plant communication increase, so might our understanding of how plants organize, communicate and thrive in stressful environments.

## Materials and methods

### Plant materials

*Arabidopsis thaliana* ecotype Columbia-0, which is publicly available from Arabidopsis Biological Resource Center (https://abrc.osu.edu/) was used as Wild type (WT). Transgenic lines GCamp3 and GCamp3/*glr3.3glr3.6* were described by Toyota et al (Toyota et al., 2018). Transgenic line *VSP1*-YFP was previously documented (Betsuyaku et al., 2018; Kinoshita and Betsuyaku, 2018; Kinoshita et al., 2019). Columbia-0 ecotype was graciously provided by Dr. Tohru Ariizumi, University of Tsukuba.

### Growth

Surface sterilized Arabidopsis seeds were grown for three weeks in Murashige and Skoog (MS) media consisting of half-strength MS, 0.5% sucrose, and 0.6% Phytagel (Kinoshita et al., 2012). Plants were grown at 22°C under 16-h/8-h light/dark cycle. After one week, seven seedlings were transferred to media inside a Petri dish divided in half by a plastic divider and grown for two additional weeks. For **Figure 1e**, 22 seedlings were transferred. Three-week-old seedlings were used in the experiments. This divider had 1 mm holes. The plastic divider in the Petri dish was manufactured at the University of Tsukuba Engineering Workshop Division.

### Insects

Armyworm (*Mythimna separata*) was reared according to Kuramitsu et al (Kuramitsu et al., 2019). Silkmate (Ehime Sanshu) was used as an artificial diet. Diamondback moths (*Plutella xylostella*) were reared using komatsuna leaves. Second and third instar armyworm larvae and fourth instar diamondback moth larvae were used.

### Microscopy

M205FA automated stereomicroscope (Leica Microsystems) with a motorized stage and DFC7000T color CCD camera (Leica Microsystems) was used to acquire images. Apparatus was controlled by LasX software (Leica Microsystems). A metal halide bulb (Leica EL6000) was used as an excitation light source. Chlorophyll auto fluorescence and YFP signals were detected using Texas Red and YFP filters respectively (both from Leica Microsystems) (Kinoshita and Betsuyaku, 2018; Kinoshita et al., 2019). Excitation emission wavelengths of YFP and Texas Red filters were 510/20–560/40 nm and 560/40–610 LP nm respectively.

In all analyzes, except **Figures 1b, c**, the first cycle was recorded without insects. Six DB moth larvae or two armyworm larvae were used in each experiment (with the exception of **Figure 1e**). Equal numbers of insects were used for control without *Emitter* (**Figures 1i**, **2d**). For the carbon dioxide control experiments, a Petri dish with *Receiver* was opened and placed in a plastic box containing approximately 800 ppm of carbon dioxide (**Figure 1h**). This environment was created by placing dry ice in the plastic box. The Petri dish was left open for one minute before being sealed and analyzed using time-lapse microscopy. To measure the effect of moisture on control experiments, we placed a cotton ball with 40 μl of ultra-pure water, which is the approximate weight of six DB moths, into a Petri dish with *Receiver* and analyzed them using time-lapse microscopy (**Figure 1h**). For experiments using our automatic fluorescent signal quantification method (Kinoshita et al., 2019), diamondback moth was added at the beginning of recordings (**Figure 1b, c**). In the experiment regarding ventilation, we used a 0.6 mm drill to drill three holes on the *Emitter* side and five holes on the *Receiver* side of the lid of the petri dish (**Supplementary Figure 3**).

Experimental research using plants and insects in our study complies with relevant international, national, and institutional rules and guidelines.

### Image processing

Red channel images from Texas Red filter and white light images were exported from LasX (Leica Microsystems). Green channel images from the YFP filter were exported and analyzed using FIJI (NIH) by superposing binarized Texas Red images to define plant shapes. These superposed images were used to extract plant-specific signals. Shadows in merged images at the intersection of the adjacent frames were adjusted using the BaSic tool (Peng et al., 2017).

### Quantification

For **Figure 1c**, the “Analysis” function of LasX was used to calculate the Texas Red area from red channel images. Mean YFP signal intensity from the whole plant was analyzed using green channel images (Kinoshita et al., 2019). To prevent the leaves from growing upward in the dark, 300 milliseconds of white light was applied for every YFP image taken on a single tile. As for the overlap between leaves or insects, 20 millisecond exposure for Texas Red imaging immediately followed five seconds of YFP exposure.

The remaining YFP signal intensity was quantified using images from the green channel with FIJI (NIH) software. For **Figures 1h, i**, fluorescent signal intensities of fully developed, non-overlapped whole leaves were measured. For **Figures 1f, g**, signal intensity at the time of Fmax was analyzed at the leaf blade closest to the petiole. Signal intensity was plotted along the horizontal orientation of a leaf using FIJI. Relative positions were used: the center of the leaf was 0.5. The edges on both ends were 0 and 1. Timing of maximum mean fluorescent signal was used for **Figures 1e, i**. For **Figure 2c**, mean signal intensity for the increased fluorescent region within midvein was compared against the rest of the leaf. For **Figures 2b, d**, length of the region of signal increase along the midvein from base of the leaf was measured using threshold of 6-94. For **Figure 2b**, data for 1.5 hours was used, for **Figure 2d**, data at 0.5 hours was used. For **Figure 1e**, acrylic box of 193 mm (W) x 103 mm (H) x 25 mm (D) was used. Box was separated into three sections using a porous divider. The first section contained five WT *Emitters*, the second section contained 15 WT Arabidopsis plants, and the third section contained two *VSP1-Receivers.* Twenty DM moths were added to the *Emitter* section after the first frame was recorded. Recording was conducted for 20-hours in one-hour intervals. Fluorescent signal intensities of fully developed, non-overlapped whole leaves were measured. The brightest two leaves were analyzed for each plant. Statistics and graphs were done using Spitzer et al. (2014) and R package (Team, R.C., 2019).

### Gas chromatography mass spectrometry

Volatile components were collected via passive absorption onto a Monotrap (RGC18 TD, GL-Science, Tokyo, Japan) for three hours at room temperature. After the volatiles were absorbed, samples were transferred to a 1.5-ml vial and stored at − 20 °C until analysis. Headspace volatiles collected using the Monotrap were analyzed by gas chromatography-mass spectrometry (GC–MS, GC: Agilent 7890A/MS5977B MSD, Agilent Technologies, CA, USA) with an HP-5MS UI capillary column (30 m, 0.25-mm ID, 0.25-μm film thickness; Agilent Technologies) equipped with a thermal-desorption system, cooled injection, and cold trap (Gerstel). The GC was maintained at 40 °C for 3 min., increased to 150 °C at a rate of 10 °C/min., then to 280 °C at 20 °C/min., and held at this temperature for five minutes. Helium was the carrier gas at a constant flow of 1.1 ml/min. The compounds were tentatively identified using data contained in the NIST Mass Spectral Library, 2017 release. GC-MS data were deconvoluted using Unknowns Analysis software (ver. B.09.00, Agilent Technologies) and aligned using Mass Profinder Professional (ver. 14.9, Agilent Technologies). Only entities present in at least 60% of replicates from one condition were included in subsequent analyzes. To determine changes in components in each condition, the data was subjected to principal component analysis using software R (version 4.1.0). To determine which components varied between conditions, we performed volcano plot analysis to compare with and without infested *Emitter* (P value 0.05, fold change 2) in the Mass Profinder Professional software. Compound identification was performed by comparing mass spectra to the NIST 2017 Library.

## Data Availability

The raw data supporting the conclusions of this article will be made available by the authors, without undue reservation.

## Supplementary material

Supplementary Figure 1 Fluorescent signals from *VSP1-Receiver* were dependent on insect damage and *GLR* genes. *VSP1-Receiver* failed to emit a clear fluorescent signal without insects (top). Armyworm-infested *Emitter* with *glr3.3/glr3.6* mutations did not induce distinct fluorescent signals in *VSP1-Receiver* (bottom). Scale bar, 5 mm.

Supplementary Figure 2 Insect damage in *Emitter* was measured using auto fluorescent imaging. Insect damage was visualized using chlorophyll autofluorescence using a Texas Red filter. Auto fluorescent images were used to quantify insect damage (**Figure 1b**). Scale bar, 1 cm.

Supplementary Figure 3 Plant communication under DB moth attack is detected under ventilated conditions. Left, Fluorescence from VSP1-*Receiver* without ventilation. Right, Fluorescence from VSP1-*Receiver* with ventilation.

Supplementary Figure 4 GLR mutant Gcamp3-*Receiver* did not respond to WT *Emitter. Gcamp3-Receiver* with *glr3.3* and *glr3.6* mutations was exposed to WT *Emitter* infested with diamondback moth (DB moth) and armyworm. *Receivers* with double mutations failed to display noticeable fluorescent signals. Scale bar, 1 mm.

Supplementary Figure 5 *Gcamp3-Receiver* does not display an increase of [Ca2+lcyt area in the midvein when exposed to infested *Emitter* with *glr3.3/glr3.6* mutations scale bar, 1 mm.

Movie S1 Exposure to DB moth-damaged *Emitter* triggers fluorescent signal in *VSP1-Receiver.* Scale bar, 5 mm. (corresponding to **Figure 1b** top panel).

Movie S2 Exposure to DB moth-damaged *Emitter* triggers fluorescent signal in *VSP1-Receiver*. first frame is recorded without DB moth. Scale bar: 5 mm. (corresponding to **Figure 1d**).

Movie S3 Exposure to armyworm-damaged *Emitter* triggers fluorescent signal in *VSP1-Receiver.* first frame is recorded without armyworm. Scale bar: 5 mm. (corresponding to **Figure 1d**).

Movie S4 Movement of [Ca^2+^]_cyt_ increase extends toward the tip of the leaf after exposure to DB moth-damaged *Emitter. Receiver* [Ca^2+^]_cyt_ was visualized using transgenic Arabidopsis expressing GCaMP3, a protein-based fluorescent calcium sensor. Scale bar: 5 mm. (corresponding to **Figure 2a**).

Movie S5 Movement of [Ca^2+^]_cyt_ increase extends toward the tip of the leaf after exposure to armyworm-damaged *Emitter. Receiver* [Ca^2+^]_cyt_ was visualized using transgenic Arabidopsis expressing GCaMP3, a protein-based fluorescent calcium sensor. Scale bar: 5 mm. (corresponding to **Figure 2a**).

## References

[B14] Aratani Y. Uemura T. Hagihara T. Matsui K. Toyota M. (2023). Green leaf volatile sensory calcium transduction in Arabidopsis. Nat. Commun. 14, 6236. doi: 10.1038/s41467-023-41589-9 37848440 PMC10582025

[B24a] Arimura G. Ozawa R. Shimoda T. Nishioka T. Boland W. Takabayashi J. (2000). Herbivory-induced volatiles elicit defence genes in lima bean leaves. Nature 406 (6795), 512–515. 10952311 10.1038/35020072

[B24] Arimura G. Uemura T. (2024). Cracking the plant VOC sensing code and its practical applications. Trends Plant Sci. doi: 10.1016/j.tplants.2024.09.005 39395880

[B11] Berger S. Mitchell-Olds T. Stotz H. U. (2002). Local and differential control of vegetative storage protein expression in response to herbivore damage in Arabidopsis thaliana. Physiol. Plant 114, 85–91. doi: 10.1046/j.0031-9317.2001.1140112.x 11982938

[B16] Betsuyaku S. Katou S. Takebayashi Y. Sakakibara H. Nomura N. Fukuda H. (2018). Salicylic acid and jasmonic acid pathways are activated in spatially different domains around the infection site during effector-triggered immunity in Arabidopsis thaliana. Plant Cell Physiol. 59, 439. doi: 10.1093/pcp/pcx181 29365197 PMC5914353

[B27] Birkett M. A. Chamberlain K. Guerrieri E. Pickett J. A. Wadhams L. J. Yasuda T. (2003). Volatiles from whitefly-infested plants elicit a host-locating response in the parasitoid, Encarsia formosa. J. Chem. Ecol. 29, 1589–1600. doi: 10.1023/a:1024218729423 12921437

[B1] Brosset A. Blande J. D. (2021). Volatile-mediated plant–plant interactions: volatile organic compounds as modulators of receiver plant defence, growth, and reproduction. J. Exp. Bot. 73, 511–528. doi: 10.1093/jxb/erab487 34791168 PMC8757495

[B20] Caldwell E. Read J. Sanson G. D. (2015). Which leaf mechanical traits correlate with insect herbivory among feeding guilds? Ann. Bot. 117, 349–361. doi: 10.1093/aob/mcv178 26715468 PMC4724051

[B33] Chen Y. Mao J. Reynolds O. L. Chen W. He W. You M. . (2020). Alyssum (Lobularia maritima) selectively attracts and enhances the performance of Cotesia vestalis, a parasitoid of Plutella xylostella. Sci. Rep. 10, 6447. doi: 10.1038/s41598-020-62021-y 32296099 PMC7160144

[B19] Duan H. Huang M. Y. Palacio K. Schuler M. A. (2005). Variations in CYP74B2 (hydroperoxide lyase) gene expression differentially affect hexenal signaling in the Columbia and Landsberg erecta ecotypes of Arabidopsis. Plant Physiol. 139, 1529–1544. doi: 10.1104/pp.105.067249 16258015 PMC1283787

[B4] Erb M. Reymond P. (2019). Molecular interactions between plants and insect herbivores. Annu. Rev. Plant Biol. 70. doi: 10.1146/annurev-arplant-050718-095910 30786233

[B31] Errard A. Ulrichs C. Kühne S. Mewis I. Drungowski M. Schreiner M. . (2015). Single- versus multiple-pest infestation affects differently the biochemistry of tomato (Solanum lycopersicum 'Ailsa Craig'). J. Agric. Food. Chem. 63, 10103–10111. doi: 10.1021/acs.jafc.5b03884 26507319

[B28] Gokila G. Premalatha K. Shanmugam P. S. Suganya Kanna S. Pradeep S. (2024). Herbivore-induced plant volatiles in rice: a natural defense mechanism shaping arthropod community. Appl. Ecol. Environ. Res. 22, 3047–3058. doi: 10.15666/aeer/2204_30473058

[B7] Gong Q. Wang Y. He L. Huang F. Zhang D. Wang Y. . (2023). Molecular basis of methyl-salicylate-mediated plant airborne defence. Nature 622, 139–148. doi: 10.1038/s41586-023-06533-3 37704724

[B22] Hu L. Ye M. Erb M. (2019). Integration of two herbivore-induced plant volatiles results in synergistic effects on plant defence and resistance. Plant Cell Environ. 42, 959–971. doi: 10.1111/pce.13443 30195252 PMC6392123

[B8] Karban R. (2021). Plant communication. Annu. Rev. Ecol. Evol. Syst. 52, 1–24. doi: 10.1146/annurev-ecolsys-010421-020045 41139587

[B6] Karban R. Shiojiri K. Huntzinger M. McCall A. C. (2006). Damage-induced resistance in sagebrush: volatiles are key to intra- and interplant communication. Ecology 87, 922–930. doi: 10.1890/0012-9658(2006)87[922:drisva]2.0.co;2 16676536

[B15] Karban R. Shiojiri K. Ishizaki S. Wetzel W. C. Evans R. Y. (2013). Kin recognition affects plant communication and defence. Proc. Biol. Sci. 280, 20123062. doi: 10.1098/rspb.2012.3062 23407838 PMC3574382

[B5] Karban R. Yang L. H. Edwards K. F. (2014). Volatile communication between plants that affects herbivory: a meta-analysis. Ecol. Lett. 17, 44–52. doi: 10.1111/ele.12205 24165497

[B17] Kinoshita N. Betsuyaku S. (2018). The effects of Lepidopteran oral secretion on plant wounds: a case study on the interaction between. Plant Bio/Technol. (Tokyo). 35, 237–242. doi: 10.5511/plantbiotechnology.18.0528a 31819728 PMC6879372

[B37] Kinoshita N. Wang H. Kasahara H. Liu J. Macpherson C. Machida K. . (2012). IAA-Ala Resistant3, an evolutionarily conserved target of miR167, mediates Arabidopsis root architecture changes during high osmotic stress. Plant Cell 24, 3590–3602. doi: 10.1105/tpc.112.097006 22960911 PMC3480289

[B18] Kinoshita N. Sugita A. Lustig B. Betsuyaku S. Fujikawa T. Morishita T. (2019). Automating measurements of fluorescent signals in freely moving plant leaf specimens. Plant Bio/Technol. (Tokyo). 36, 7–11. doi: 10.5511/plantbiotechnology.18.1002a 31275043 PMC6566008

[B38] Kuramitsu K. Vicencio E. J. M. Kainoh Y. (2019). Differences in food plant species of the polyphagous herbivore Mythimna separata (Lepidoptera: Noctuidae) influence host searching behavior of its larval parasitoid, Cotesia kariyai (Hymenoptera: Braconidae). Arthropod-Plant. Interact. 13, 49–55. doi: 10.1007/s11829-018-9659-0

[B36] Li C. Schilmiller A. L. Liu G. Lee G. I. Jayanty S. Sageman C. . (2005). Role of beta-oxidation in jasmonate biosynthesis and systemic wound signaling in tomato. Plant Cell 17, 971–986. doi: 10.1105/tpc.104.029108 15722469 PMC1069712

[B35] Liang X. Qian R. Wang D. Liu L. Sun C. Lin X. (2022). Lipid-derived aldehydes: new key mediators of plant growth and stress responses. Biology 11, 1590. doi: 10.3390/biology11111590 36358291 PMC9687549

[B9] Mousavi S. A. Chauvin A. Pascaud F. Kellenberger S. Farmer E. E. (2013). GLUTAMATE RECEPTOR-LIKE genes mediate leaf-to-leaf wound signalling. Nature 500, 422–426. doi: 10.1038/nature12478 23969459

[B25] Nagashima A. . (2019). Transcriptional regulators involved in responses to volatile organic compounds in plants. J. Biol. Chem. 294, 2256–2266. doi: 10.1074/jbc.ra118.005843 30593507 PMC6378981

[B23] Nguyen C. T. Kurenda A. Stolz S. Chételat A. Farmer E. E. (2018). Identification of cell populations necessary for leaf-to-leaf electrical signaling in a wounded plant. Proc. Natl. Acad. Sci. U.S.A. 115, 10178–10183. doi: 10.1073/pnas.1807049115 30228123 PMC6176584

[B39] Peng T. Thorn K. Schroeder T. Wang L. Theis F. J. Marr C. . (2017). A BaSiC tool for background and shading correction of optical microscopy images. Nat. Commun. 8, 14836. doi: 10.1038/ncomms14836 28594001 PMC5472168

[B2] Schuman M. C. Baldwin I. T. (2016). The layers of plant responses to insect herbivores. Annu. Rev. Entomol. 61, 373–394. doi: 10.1146/annurev-ento-010715-023851 26651543

[B40] Spitzer M. Wildenhain J. Rappsilber J. Tyers M. (2014). BoxPlotR: a web tool for generation of box plots. Nat. Methods 11, 121–122. doi: 10.1038/nmeth.2811 24481215 PMC3930876

[B41] Team, R.C . (2019). R: Language and environment for statistical computing (Vienna, Australia: Foundation for Statistical Computing). Available online at: https://www.R-project.org/ (Accessed May 28, 2018).

[B12] Toyota M. Spencer D. Sawai-Toyota S. Jiaqi W. Zhang T. Koo A. J. . (2018). Glutamate triggers long-distance, calcium-based plant defense signaling. Science 361, 1112–1115. doi: 10.1126/science.aat7744 30213912

[B3] Turlings T. C. J. Erb M. (2018). Tritrophic interactions mediated by herbivore-induced plant volatiles: mechanisms, ecological relevance, and application potential. Annu. Rev. Entomol. 63, 433–452. doi: 10.1146/annurev-ento-020117-043507 29324043

[B21] Wang L. Erb M. (2022). Volatile uptake, transport, perception, and signaling shape a plant's nose. Essays. Biochem. doi: 10.1042/ebc20210092 36062590 PMC9528081

[B26] Winter D. Vinegar B. Nahal H. Ammar R. Wilson G. V. Provart N. J. (2007). An "Electronic Fluorescent Pictograph" browser for exploring and analyzing large-scale biological data sets. PloS One 2, e718. doi: 10.1371/journal.pone.0000718 17684564 PMC1934936

[B29] Yasa V. Suroshe S. S. Nebapure S. M. (2024). Behavioral response of zigzag ladybird beetle Cheilomenes sexmaculata to the HIPVs induced by cotton aphid, Aphis gossypii. Arthropod-Plant. Interact. 18, 771–780. doi: 10.1007/s11829-024-10087-0 30311153

[B30] Yi C. Teng D. Xie J. Tang H. Zhao D. Liu X. . (2023). Volatiles from cotton aphid (Aphis gossypii) infested plants attract the natural enemy Hippodamia variegata. Front. Plant Sci. 14. doi: 10.3389/fpls.2023.1326630 38173929 PMC10761428

[B34] Zhai R. Zhang H. Xie Y. Zhang S. Zhou F. Du X. . (2025). Naturally impaired side-chain shortening of aromatic 3-ketoacyl-CoAs reveals the biosynthetic pathway of plant acetophenones. Nat. Plants 11, 1903–1919. doi: 10.1038/s41477-025-02082-x 40913079

[B10] Zhang L. Zhang F. Melotto M. Yao J. He S. Y. (2017). Jasmonate signaling and manipulation by pathogens and insects. J. Exp. Bot. 68, 1371–1385. doi: 10.1093/jxb/erw478 28069779 PMC6075518

[B32] Zubkov F. I. Kouznetsov V. V. (2023). Traveling across life sciences with acetophenone-a simple ketone that has special multipurpose missions. Molecules 28. doi: 10.3390/molecules28010370 36615564 PMC9823374

