# Supplementary figures and images for: Revisiting volatile organic compounds’ role in plant communication using real-time bioimaging
Source: Front Plant Sci. 2026 Jun 18;17:1829872. doi: 10.3389/fpls.2026.1829872 (PMC13323021; doi:10.3389/fpls.2026.1829872)

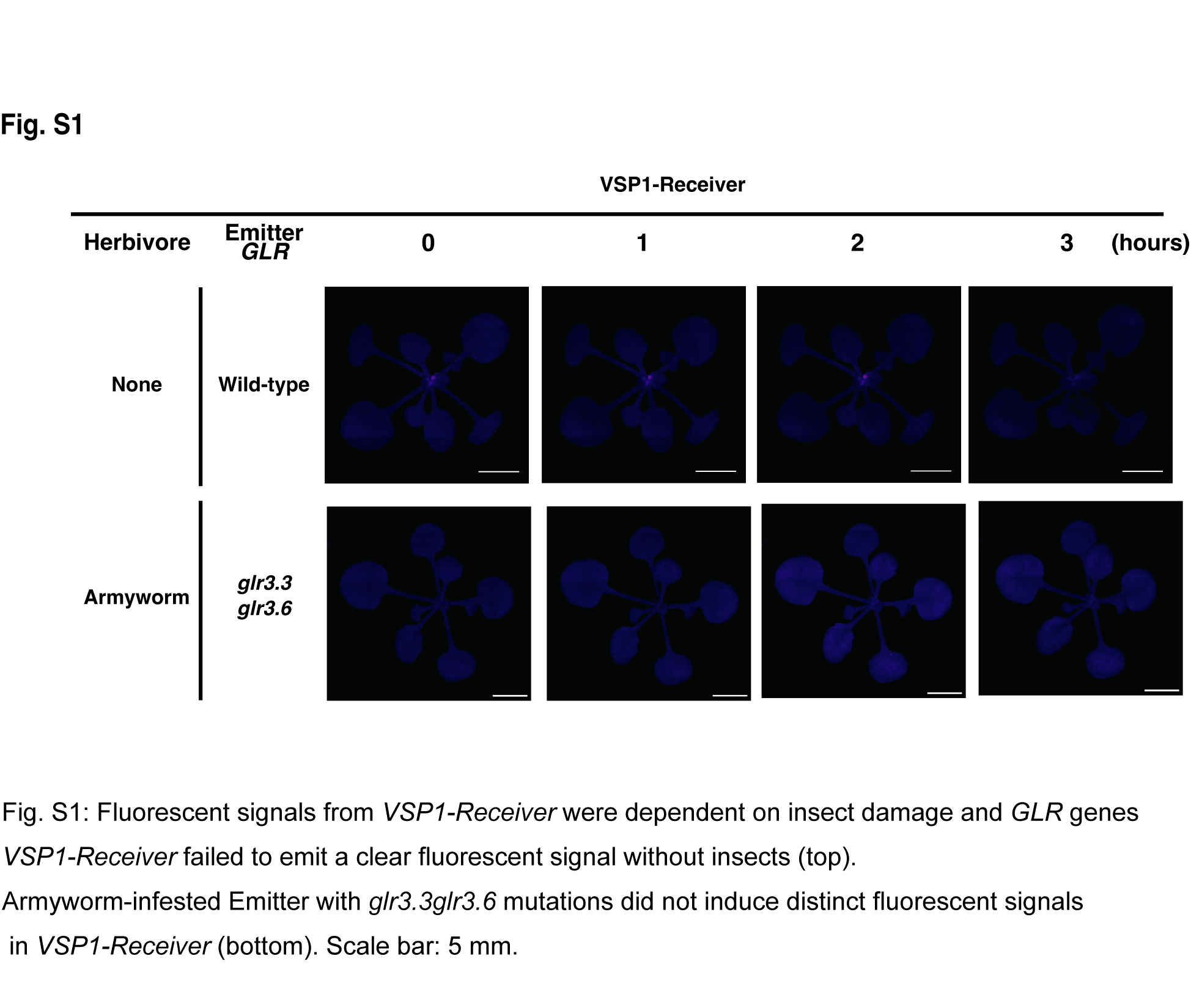

Supplement: Supplementary Figure 1 — Fluorescent signals from VSP1-Receiver were dependent on insect damage and GLR genes. VSP1-Receiver failed to emit a clear fluorescent signal without insects (top). Armyworm-infested Emitter with glr3.3/glr3.6 mutations did not induce distinct fluorescent signals in VSP1-Receiver (bottom). Scale bar, 5 mm. [file Image1.tif]

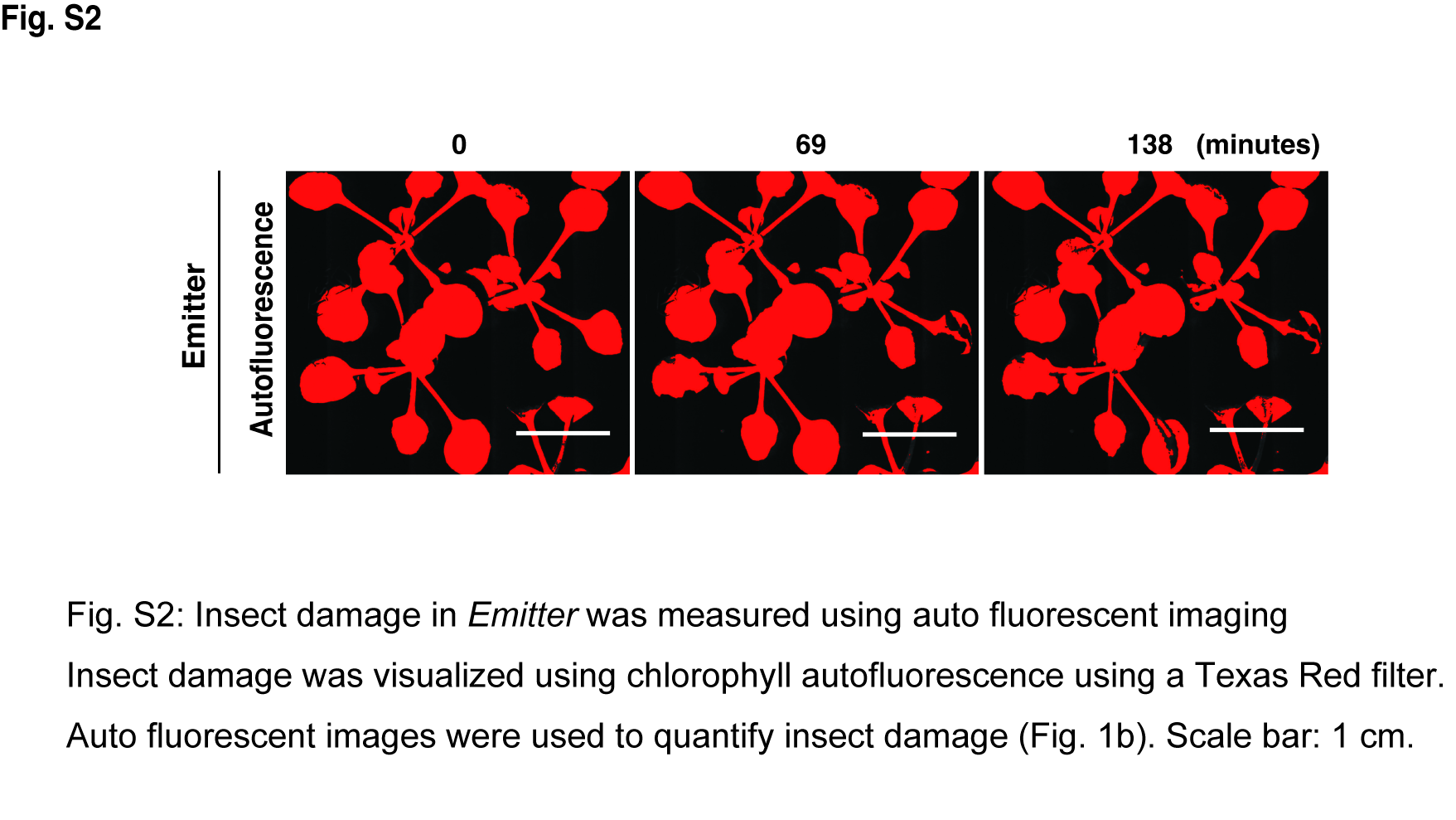

Supplement: Supplementary Figure 2 — Insect damage in Emitter was measured using auto fluorescent imaging. Insect damage was visualized using chlorophyll autofluorescence using a Texas Red filter. Auto fluorescent images were used to quantify insect damage (Figure 1b). Scale bar, 1 cm. [file Image2.tif]

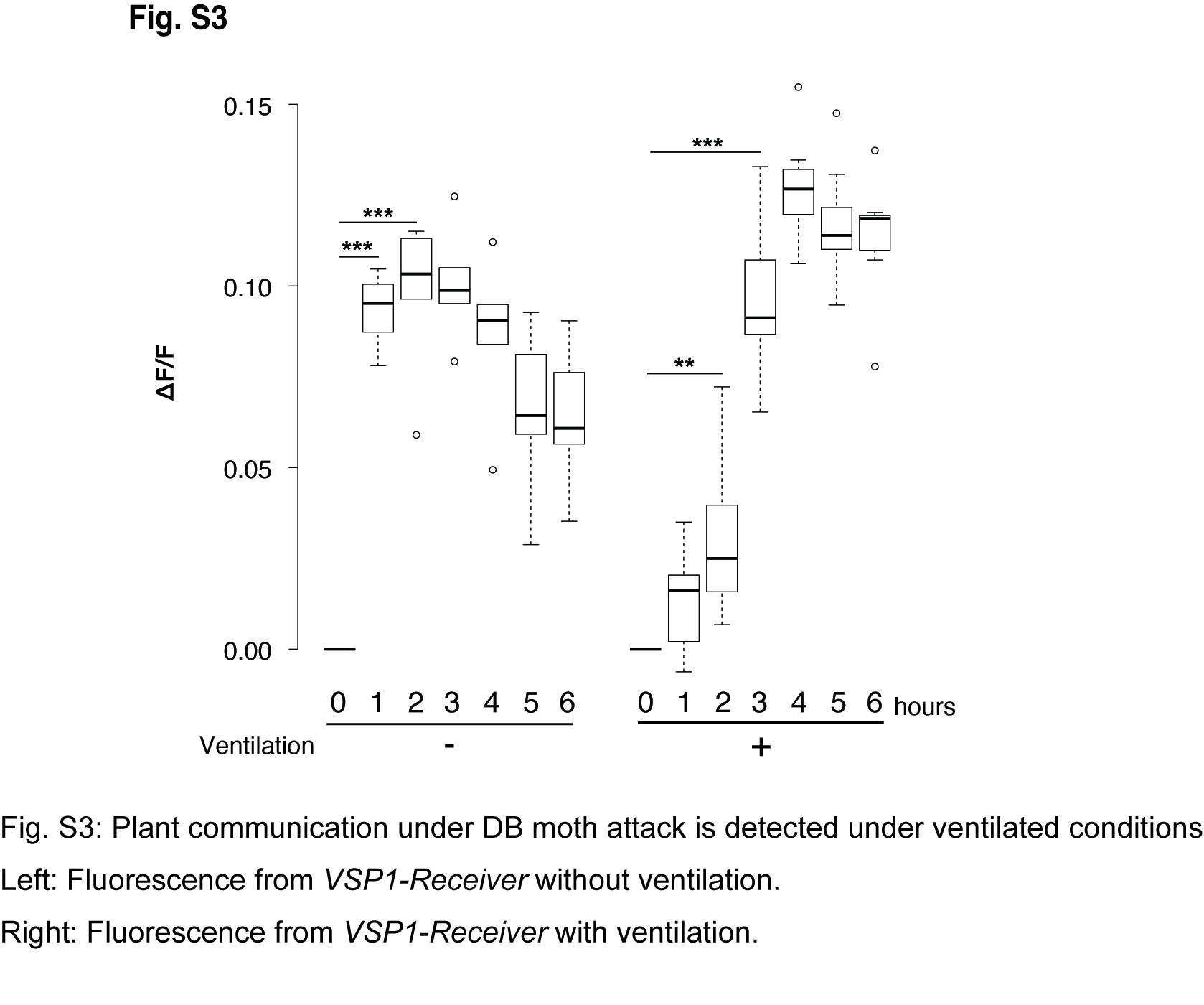

Supplement: Supplementary Figure 3 — Plant communication under DB moth attack is detected under ventilated conditions. Left, Fluorescence from VSP1-Receiver without ventilation. Right, Fluorescence from VSP1-Receiver with ventilation. [file Image3.tif]

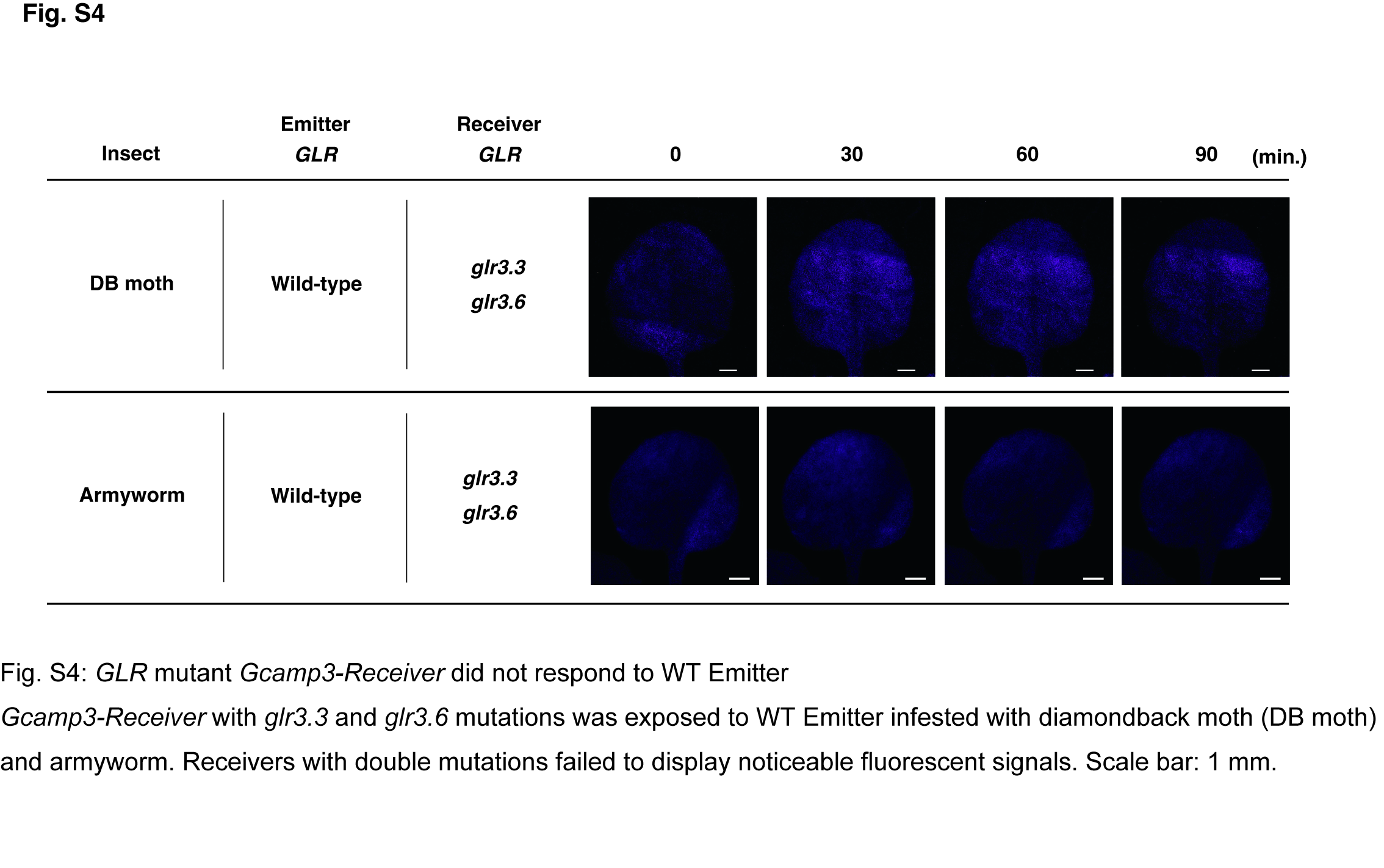

Supplement: Supplementary Figure 4 — GLR mutant Gcamp3-Receiver did not respond to WT Emitter. Gcamp3-Receiver with glr3.3 and glr3.6 mutations was exposed to WT Emitter infested with diamondback moth (DB moth) and armyworm. Receivers with double mutations failed to display noticeable fluorescent signals. Scale bar, 1 mm. [file Image4.tif]

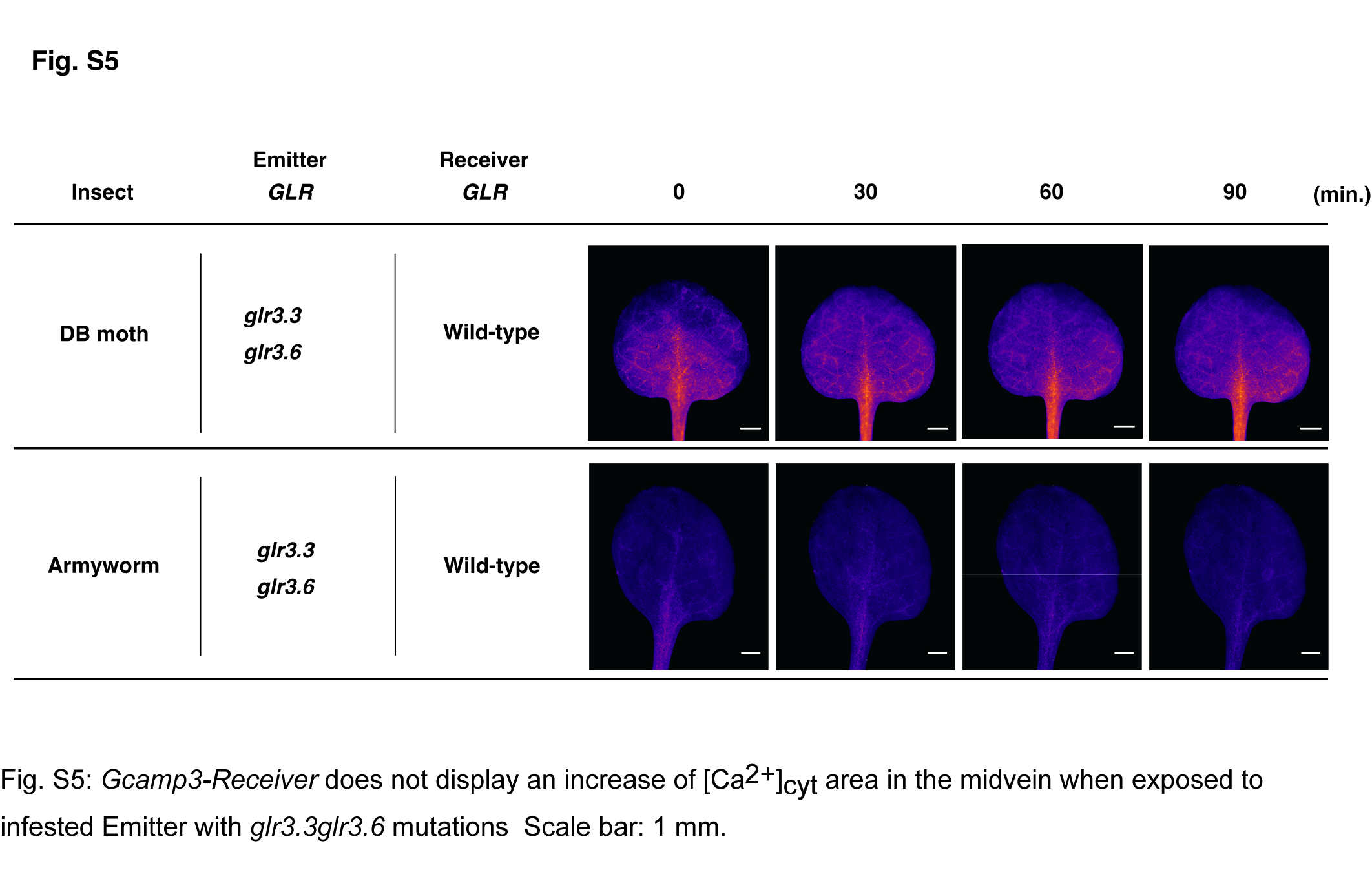

Supplement: Supplementary Figure 5 — Gcamp3-Receiver does not display an increase of [Ca2+lcyt area in the midvein when exposed to infested Emitter with glr3.3/glr3.6 mutations scale bar, 1 mm. [file Image5.tif]

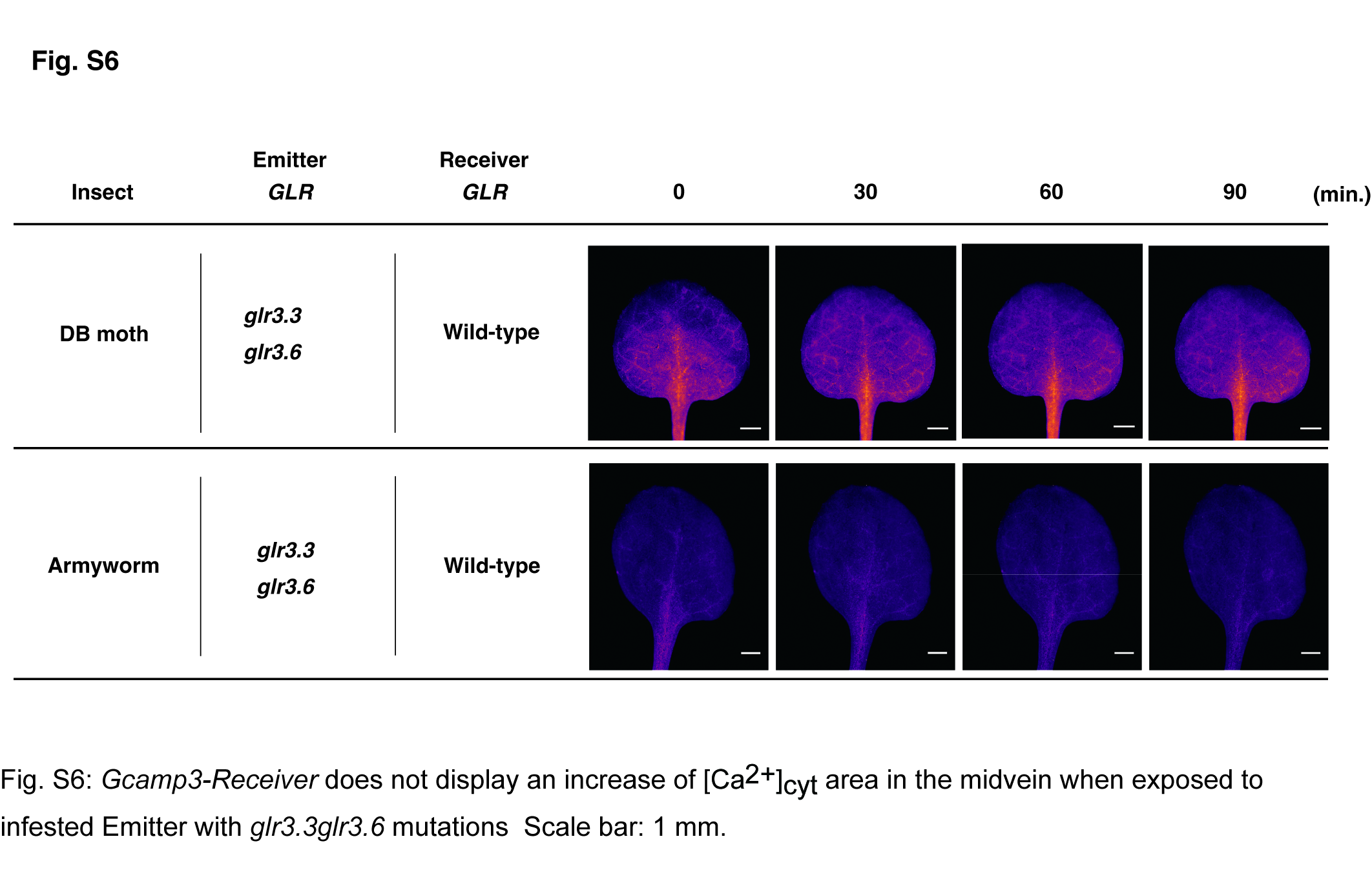

Supplement: Supplementary file 13 [file Image6.tif]
